# Primary malignant melanoma of the oesophagus: a case report

**DOI:** 10.1186/1752-1947-1-50

**Published:** 2007-07-14

**Authors:** Justin Kelly, Mary Leader, Patrick Broe

**Affiliations:** 1 Department of General Surgery, Beaumont Hospital, Dublin 9, Ireland; 2 Department of Pathology, Beaumont Hospital, Dublin 9, Ireland

## Abstract

Primary malignant melanoma of the oesophagus is a rare neoplasm comprising less than 0.2% of all primary oesophageal neoplasms. There are fewer than 250 reported cases in worldwide literature. Several reports suggest that it has a mean survival rate of 2.2% at 5 years and a median survival rate of 10 months. A 48 year old male presented to our surgical service complaining of a three month history of progressively worsening dysphagia with associated regurgitation and unintentional weight loss of 14 kg. There was no prior history of cutaneous or ocular melanoma. He was treated with a combination of subtotal oesophageal resection and immunomodulatory therapy. We present herein a case of primary malignant melanoma of the oesophagus including the associated clinical, pathological and radiological findings.

## Case presentation

A previously healthy 48 year old male presented to our surgical out-patient service complaining of a 3 month history of progressively worsening dysphagia for solids with associated regurgitation and unintentional weight loss of 14 kg. Physical examination was unremarkable and there was no evidence of organomegaly or lymphadenopathy. Subsequent oesophagoscopy revealed a large polypoid pigmented lesion at 30 cm. The lesion did not impede the passage of the scope. Multiple biopsies were taken. Two pigmented cutaneous lesions (no sinister features present in either lesion) were also excised – histology showed benign lesions. No evidence of melanoma was found.

A staging CT scan of his thorax, abdomen and pelvis showed a well-defined eccentric mass in the mid-lower oesophagus. There was no apparent local invasion or regional lymphadenopathy.

**Figure 1:**
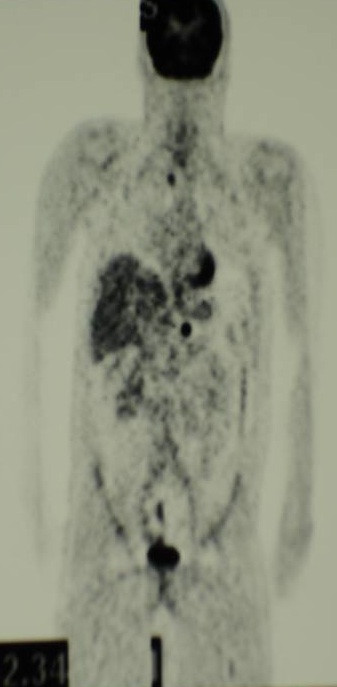
FDG PET CT.

Whole body FDG PET/CT body scan confirmed the mass in the oesophagus with increased uptake in a high abdominal pre-aortic and high right paratracheal node consistent with metastasis. (see figure 1).

**Figure 2:**
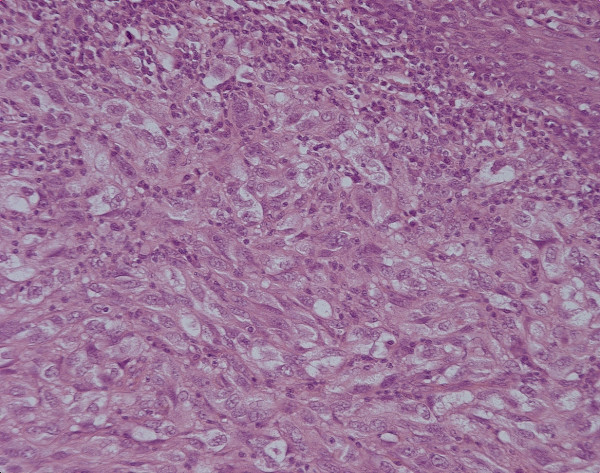
high power H & E stain and S100 stain of biopsy.

**Figure 3:**
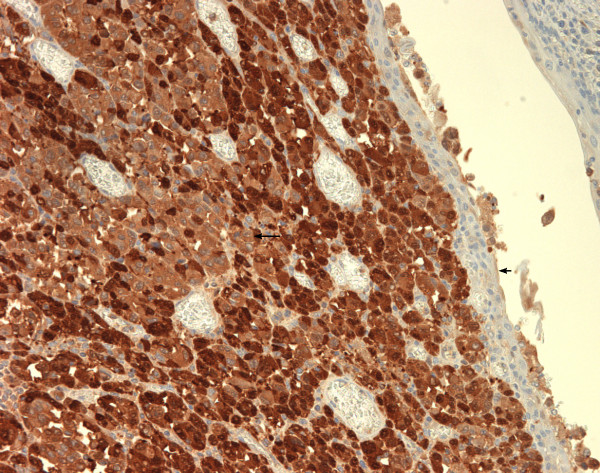
high power H & E stain and S100 stain of biopsy.

Biopsy results from the oesophagoscopy showed an infiltrating malignant tumour with prominent nucleoli and cells prominent in the submucosa and also in the basal layer of the squamous mucosa. S100 stain was positive. Features were consistent with malignant melanoma. The features that confirmed the primary nature of the neoplasm were the junctional change, multi-pleomorphic spindle shaped cells with prominent nucleoli and some lymphocytic infiltrate. (see figures 2 &3).

The absence of cutaneous, ocular, or mucosal melanoma elsewhere also supported a diagnosis of primary rather than secondary melanoma.

He underwent a three stage oesophagectomy with mediastinal lymphadenectomy. Seven of the twenty six lymph nodes were postitive for melanoma, including the two nodes highlighted by the FDG-PET scan. The remainder of his post-operative stay was unremarkable and he was discharged home.

He underwent a course of immunomodulatory therapy, consisting of a 4 week course of daily high dose IV Interferon alpha 2b and then received a thrice weekly lower dose subcutaneous regimen for a further 48 weeks. He tolerated this regimen well. He was seen regularly in the immediate follow up period and, at his 18 months post-operative review, there was no evidence of disease recurrence and he continued to do well.

## Discussion

According to the National Cancer Registry of Ireland [1] there are approximately 300 new cases of oesophageal carcinoma diagnosed each year, the majority being adenocarcinoma, with a male:female ratio of 2:1. Classical risk factors include Barrett's oesophagitis, smoking, alcohol, familial preponderance and dietary factors.

Primary oesophageal melanoma is an extremely rare non-epithelial neoplasm, accounting for less than 0.2% of all primary oesophageal neoplasms with less than 250 cases reported worldwide [2]. It commonly presents in a similar manner to other oesophageal malignancies. The mean survival rate is reputed to be less than 5% at 5 years and a median survival rate of ten months [3] with a disease related mortality of 85% [4]. 90% of cases occur in the middle or distal third of the esophagus, usually as a solitary tumor, but multiple lesions have been reported in 12% of cases [3].

Gross appearances are typically those of a polypoid, intraluminal mass which may, or may not be obstructive. 85% of lesions are pigmented. However, numerous cases of amelanotic melanoma of the oesophagus have been reported. Microscopically, it usually involves the mucosal and submucosal layers, growing in a lentiginous radial manner. Lymphovascular space invasion is common. Histologically melanoma is composed of epithelioid cells arranged in nests or spidle cells arranged in fasicles, with or without melanin deposition of melanin pigment. If a tumour is amelanotic, it may be difficult to recognise as malignant melanoma without ancillary immunohistochemical staining [2].

At the time of presentation, metastatic disease is present in approximately 50% of patients, 31% hepatic, 29% mediastinal, 18% pulmonary, and 13% cerebral [3].

Other sites of primary melanoma must be excluded [4]. The Breslow thickness of tumour invasion for cutaneous melanoma is a good predictor of outcome. However, given that primary melanoma of the oesophagus is so rare, it is difficult to apply this staging tool in this setting.

The principles involved in staging any histological type of oesophageal malignancy are to distinguish between locoregional and systemic disease, to assess the extension of local disease and to determine the possible response to neo-adjuvant therapy.

Flurodeoxyglucose positron emission tomography (FDG PET/CT) has proved to be an excellent method for staging of metastatic melanoma. Due to its high sensitivity for malignant lesions and the possibility of covering the whole body in one examination, it can supplement other staging tools.

Because of the high tumour-to-background ratio, FDG-PET can highlight metastases at unusual sites that are missed with conventional imaging modalities.

Furthermore, it provides information on the malignant potential of the detected lesion. Given the relative scarcity of primary melanomas of the oesophagus little is known about its application for these tumours [5,6].

Ott et al recently reported that in oesophageal cancer FDG PET/CT has been shown to detect metastatic disease in approximately 20% of patients who are considered as having only locoregional disease on CT, similar to the patient reported in this case. The sensitivity of computed tomography (CT) for detection of distant metastases ranges between <50% and >90%. They reported a specificity of 80% for locoregional pretherapeutic tumour staging [7].

Classical histological appearances are seen with tumour marker staining. In the diagnosis of cutaneous melanoma many specialized immunohistochemical stains may be applied. S-100 staining has 95% sensitivity for melanoma. HMB-45 staining is used for detecting active melanocytes.

Primary treatment is surgical excision with discretionary lymphadenectomy for operable melanomas, but total or near-total oesophagectomy offers the best survival outcome (about 5 years, versus 9 months for local resection) [4]. Additional immunomodulatory therapy may be used if there is evidence of metastatic disease.

Adjuavant or neoadjuvant radiotherapy has been used but its utility is unproven [8].

Interferon alpha is used in a range of neoplastic conditions, including renal cell carcinoma, cutaneous melanoma and chronic myeloid leukaemia. It stimulates humoral and cell mediated response and thus has anti-proliferative effects. However it is not curative. Well known side effects to this therapy exist include flu-like symptoms and fatigue but fortunately our patient tolerated his treatment well.

## Competing interests

The author(s) declare that they have no competing interests.

## Authors' contributions

JK collected all the included data, conceived of the study, carried out detailed literature review and coordinated the multi disciplinary approach to the manuscript.

PB oversaw all aspects of the reports' design and helped to draft the manuscript.

ML carried out and reported on this rare histopathological diagnosis.

All authors read and approved the final manuscript
